# A Unified Approach to Analysis of Body Condition in Green Toads

**DOI:** 10.3390/d15010043

**Published:** 2022-12-23

**Authors:** Lukas Landler, Stephan Burgstaller, Magdalena Spießberger, Andras Horvath, Zhivko Zhelev, Ivelin Mollov, Ulrich Sinsch, Johannes Nepita, Florian Schwabel, Wolfgang Kuhn, Christian Köbele, Heinz Sedlmeier, Cornelia Amon, Joanna Mazgajska, Tomasz D. Mazgajski, Amir Sistani, Rieke Schluckebier, Eberhard Andrä, Moritz Ott, Günter Gollmann

**Affiliations:** 1 Institute of Zoology, University of Natural Resources and Life Sciences (BOKU), Gregor-Mendel-Straße 33, 1180 Vienna, Austria; 2 Department of Evolutionary Biology, University of Vienna, Djerassiplatz 1, 1030 Vienna, Austria; 3 Department of Game Management and Wildlife Biology, Faculty of Forestry and Wood Sciences, Czech University of Life Sciences Prague, Kamýcká 129, 165 00 Prague, Czech Republic; 4 Department of Human Anatomy and Physiology, Faculty of Biology, University of Plovdiv “Paisii Hilendarski”, 24 Tzar Assen Str., 4000 Plovdiv, Bulgaria; 5 Department of Ecology and Environmental Conservation, Faculty of Biology, University of Plovdiv “Paisii Hilendarski”, 24 Tzar Assen Str., 4000 Plovdiv, Bulgaria; 6 Department of Biology, AG Zoology, Institute of Integrated Sciences, University of Koblenz-Landau, 56070 Koblenz, Germany; 7 Wissenschaftszentrum Weihenstephan für Ernährung, Landnutzung und Umwelt der Technischen Universität München, Alte Akademie 8, 85354 Freising, Germany; 8 Landesbund für Vogelschutz in Bayern e.V. (LBV), Kreisgruppe München, Klenzestr. 37, 80469 München, Germany; 9 Museum and Institute of Zoology, Polish Academy of Sciences, Wilcza 64, 00-679 Warszawa, Poland; 10 NABU—Naturschutzstation Leverkusen-Köln, Friedrich-Ebert-Str. 49, 50996 Köln, Germany; 11 Independent Researcher, Ebenreuth 47, 94169 Thurmansbang, Germany; 12 Landschaftserhaltungsverband Landkreis Ravensburg e.V., Frauenstr. 4, 88212 Ravensburg, Germany

**Keywords:** amphibia, biometry, conservation, life history, population ecology

## Abstract

Body condition is increasingly used to assess the status of populations and as a proxy for individual fitness. A common, quick and non-invasive approach is to estimate condition from the relation between body length and mass. Among the methods developed for this purpose, the Scaled Mass Index (SMI) appears best suited for comparisons among populations. We assembled data from 17 populations of European green toads (Bufotes viridis) with the aim of devising a standard formula applicable for monitoring this species. The mean value of the exponents describing length–mass allometry in these samples was 3.0047. Hence, we propose using 3 as a scaling coefficient for calculating the SMI in green toads. From the contrast of SMI values for both sexes within populations, estimated with either the population-specific or the standard coefficient, we conclude that applying the standard formula not only facilitates comparisons among populations but may also help to avoid misinterpretation of variation within populations.

## 1 Introduction

The body condition has received increasing attention in amphibian ecology, especially in the context of conservation. It can be defined as the nutritional state or amount of energy stores of an animal and is often regarded as a measure or proxy of fitness [1]. For instance, body conditions can be used to measure environmental as well as anthropogenic effects on amphibian individuals and populations and might be used to detect species declines and habitat needs [2–4]. Assessing the condition from the relation of body mass to a measure of body length, usually snout-vent length in anurans, is a quick and non-invasive method. Various condition indices have been developed for this purpose [2,5]. We do not intend to provide a full review of these methods here but focus on a pragmatic approach to allow comparisons among different populations.

A widely applied approach uses residuals of ordinary least square (OLS) regression of mass on length [6,7]. For our aim, this residual index is out of question, as its values only make sense within the context of a certain dataset and would not allow a population comparison between two publications. In recent years, the scaled mass index (SMI) proposed by Peig and Green [5,8] has been increasingly employed in amphibian ecology. Following the feeding trials by MacCracken and Stebbings [9] it was utilized in a variety of studies, both in the field and in experimental settings [10–14].

One advantage of SMI is that the values can readily be compared among different populations of the same species [8]. To fully realize this advantage, however, researchers have to use the same formula with identical parameters for calculating the index. It seems to be common practice now that, for each project, reference lengths and scaling exponents for computing the SMI are newly determined. Thus, comparisons between results would require further tedious calculations, possibly only if scaling exponents and reference lengths were reported in all publications.

Here, we assemble and analyze data from 17 populations of European green toads, *Bufotes viridis*, to underpin a proposal for a unified approach to measuring body condition. We stipulate a reference length and explore the effects of varying the scaling exponent on the results of comparisons between sexes. We conclude that using a standard exponent not only facilitates comparisons of results among different studies but may also help to avoid misinterpretation of variation within populations.

## 2 Materials and Methods

### 2.1 Study Species

The green toad *Bufotes viridis* (Laurenti, 1768) is widespread in central, eastern and southern Europe and reaches southernmost Scandinavia. It has been characterized as a ‘typical inhabitant of cultivated steppes, avoiding wood complexes’ [15], and is regarded as a pioneer species breeding in temporary water bodies that it can colonize rapidly. Owing to its synanthropic occurrence, this species is strongly affected by changes in land use; its high mobility makes it particularly susceptible to road-kills [16,17]. There are noticeable local declines, for instance, in Cologne, where the green toad populations decreased rapidly in the matter of a decade [18]. Despite the fact that green toads could be observed in the centers of many cities, Polish urban populations also face strong declines [19–21].

The taxonomy of this group has been unstable, partly due to widespread cytonuclear discordance. Dufresnes et al. [22] clarified the relationships among the major lineages, but the species rank of several taxa is still disputed. Speybroek et al. [23] suggested treating balearicus from the Apennine peninsula and Western Mediterranean islands and sitibundus as subspecies of *B. viridis* rather than distinct species. Range boundaries between *B. v. viridis* and its eastern neighbor, *B. v. sitibundus*, are currently not well defined. Our sampling is somewhat biased toward north-western populations, but covers a considerable extent of latitude, longitude and altitude in the distribution of *B. viridis*.

### 2.2 Data Acquisition

**Figure 1:**
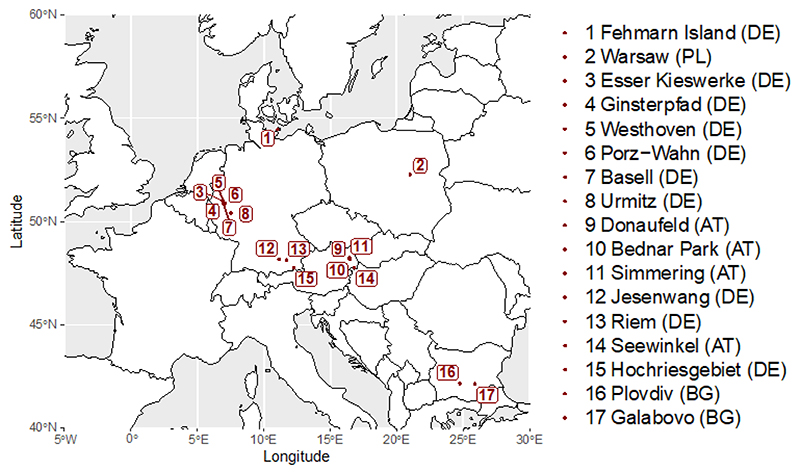
Locations of green toad populations in Europe included in this study.

Data were provided by the co-authors, originating from several different green toad monitoring projects (Figure 1). Toads were caught at or close to the breeding site, weighed, measured (snout-vent-length, SVL) and in the majority of studies sexed by external characteristics (i.e.,nuptial pad). Only sites with data fromat least 12 animals were included in the analysis.

For details of the methods, see the studies at Urmitz [24], Donaufeld [25], Hochriesgebiet [26,27], Fehmarn [28], Jesenwang [29] and Riem [30]. Investigations in Bednar Park, Seewinkel and Simmering followed the same protocol as Sistani et al. [25], protocols for Bulgarian studies (Plovdiv and Galabovo) were described in Zhelev et al.[31], the Warsaw dataset followed Mazgajska and Mazgajski [32]. For surveys in the Cologne area (sites 3 to 7, Figure 1), length was measured with sliding calipers (precision: 2.54/325.12cm ± 0.05mm) and mass was measured with a lab scale (precision 200/0.01 g). Individuals were photographed to determine recaptures.

### 2.3 Statistical Analysis

All analyses and figures were prepared using R [33]. The general approach of this index is to calculate mi = m* (L_0_/L)^b^, where mi is the (scaled) mass index (SMI), m is the individual mass, L_0_ is a reference (often “mean”) snout vent length (SVL), L is the individual’s SVL, and b is a coefficient.We followed the method described by Peig and Green [8] for calculating b using the coefficient of the standardized major axis from a linear model including the natural logarithm of mass as y and the natural logarithm of length as x (ln(m)~ln(L)). In contrast to the original paper, which used ordinary least squares (OLS), we used a robust linear model (rlm function in R); this approach is less sensitive to outliers ([34], see also Chen-Pan Liao [35] for a comparison). We used a reference length (L_0_) of 60 mm throughout. The coefficient b was calculated for each population (n = 17) and then either used directly to calculate the ‘population-specific’ SMI or used to calculate an average b for all populations, which was used as the ‘green toad coefficient’ for all individuals. This overall average b was calculated without weighting based on the sample size, as a higher number of individuals in one population should not change the mean (instead reduce random variation). In the case of several measurements for the same individual, we averaged each individual before calculating the SMI. To further explore the variation between population-specific and overall b, we calculated the difference between them and plotted it against the range of SVL variation within each sample. For comparisons between sexes, we calculated the SMI separately for females and males using the scaling exponents derived from the entire sample.

Data were summarized using the mean mass, length and SMI indices for each population with the according range and standard deviation, respectively. The map was plotted using the packages rnaturalearth and ggplot2 [36]. Predictions were calculated and plotted using the ggeffects package [37], scatterplots for each site were plotted using ggplot2 [36] and the boxplotsusing ggpubr [38], data pointswere added usinga small random error (‘jit-ter’) to reduce overplotting. The elevation data was retrieved using the elevatr package [39] and the table was created using gt [40].

## 3 Results

**Figure 2:**
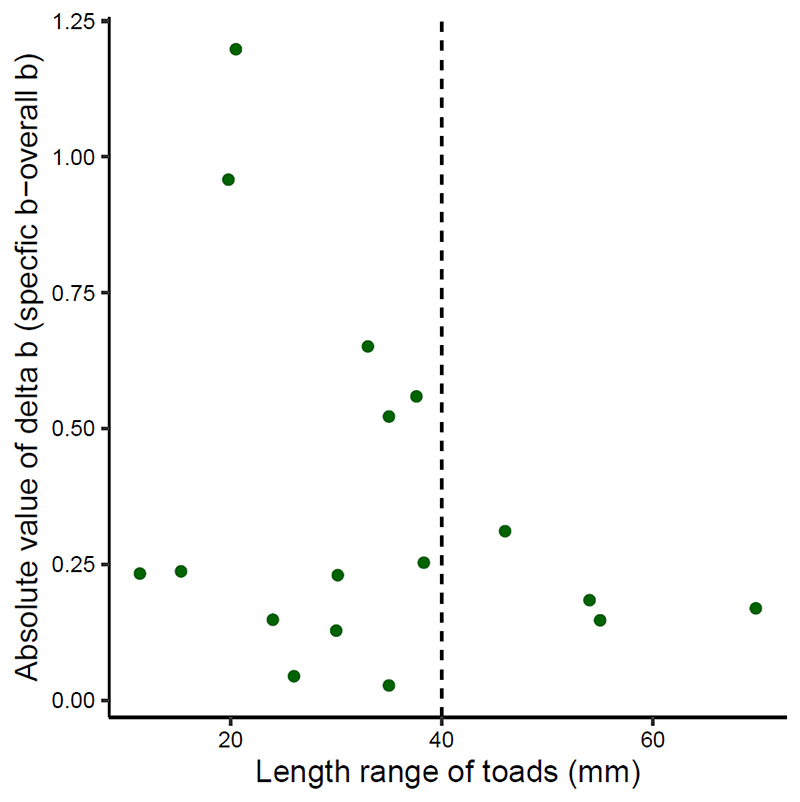
The range of toad lengths for each population plotted against the absolute differences between the green toad coefficient (overall b) and population-specific coefficient (specific b). Variations around the mean decreased with a larger length range; above 40mm (dashed line) variation remained under 0.5.

The average b across all studies was 3.0047 and we therefore used 3 as our ‘green toad coefficient’. The population-specific b varied between 2.042 and 4.198 (Table 1). The variation around the ‘green toad coefficient’ appeared to decrease with an increasing range of toad lengths, i.e., including juvenile toads as well as adults (Figure 2). We wrote an R function (“scaledMassGT”) and example R script that uses the Donaufeld dataset to showcase how this method is applied (suppl.RCode, Supplementary Materials).

**Figure 3:**
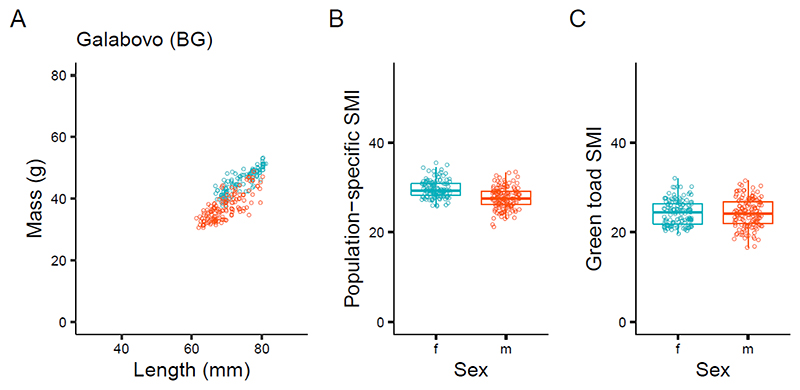
Mass versus lengths of toads in Galabovo (BG) (**A**) and the SMI of females and males using the population-specific coefficient (**B**) and the green toad coefficient (**C**). Boxplots show median (boldline), the 25th and 75th percentiles (lower and upper end of the box), as well as the 0% and 100% percentiles (lower and upper end of whiskers, excluding outliers). The reference length for SMI calculations was 60 mm and coefficient b was 2.042 (population-specific SMI) and 3 (green toad SMI).

There were no consistent differences between males and females in terms of SMI across sites, however at some sites females showed slightly higher indices. At one site (Galabovo, BG), such a difference was present using the population-specific coefficient but absent when using the green toad coefficient (Figure 3).

## 4 Discussion

Sample-specific estimates of the scaling exponent varied widely, but their average was 3, a value plausible from the first principles. It is also close to the exponents calculated by Santini et al. [41] for length-mass allometry in Anura (3.098) and Bufonidae (2.914). Therefore, we propose using 3 as a standard exponent for applying the SMI in *Bufotes viridis*. We do not claim that this value is the ‘true’ allometric exponent for all populations, but we argue that employing this average is a conservative approach. We chose 60 mm as the reference length because it is a round number in the middle of the overall size range of the total sample.

SMI can account for ontogenetic allometry and sexual dimorphism [5]. Sexual size dimorphism was present in our study populations, with females being, on average, longer and heavier than males (Figure S1). In our measure of condition, the SMI variation in both sexes was often remarkably similar across populations (Figure S1). In the two samples with the strongest deviation of population-specific b, Galabovo and Porz-Wahn, SMI values differed markedly between sexes when applying these exponents, but these differences nearly disappeared when using the standard exponent (Figure 3 and Figure S1). We infer that samples with small ranges of size variation, i.e., those consisting only of either adults or juveniles, seem to be more prone to yielding deviant estimates of the scaling exponent, failing to represent ontogenetic allometry correctly, than samples spanning a wide range of body sizes (Figure 2). Measurements of small individuals may be more affected by ‘variation’, because SVL was measured at a constant precision independent of total size, i.e., the measurement error was greater in small than in large individuals, if all were measured with the same accuracy. This measurement error, however, should not affect the slope of the regression.

For the interpretation of condition values, information about the dynamics of their variations should be particularly useful [42,43]. Sex ratios were unequal in most of our samples, owing to the skewed operational sex ratio at breeding sites. Mean SMI values for the females deviated from those of males in either direction in several populations (Figure S1). Whether females were captured and weighed before or after spawning probably explains many of these differences.With spawn deposition, females abruptly lose mass (about 30% in an Italian population [44]); the body mass of males may also decrease during breeding activity, but not so rapidly.In addition to seasonal fluctuations, the degree of stomach filling may introduce further variation, with large and heavy prey items temporarily raising body mass readings.

We caution against equating high condition values rashly with good health or high fitness. In pioneer species breeding in ephemeral water bodies, opportunities for reproduction often vary greatly among populations and seasons. High condition values probably testify to good feeding conditions, but they may also indicate a lack of occasions to invest stored resources in reproduction [45].

We refrained from attempting to analyze large-scale trends in the body condition of green toads from our results. The dataset appears too small and unbalanced for such comparisons, and we do not want to preempt conclusions from work that is still in progress. We hope, however, that our approach, including R implementation, will be useful in the entire range of our focal species. In comparisons among different studies, interobserver bias in measurements will inevitably cause some noise in the variation patterns. Nevertheless, we envisage that a unified approach to assessing body condition will yield important new insights into life-history variation in green toads.

It appears straightforward to extend this attempt to standardize the reporting of condition values to other species. We suggest that calculating the average values from several independent studies to determine the scaling exponent will reduce errors due to stochasticity. To account for ontogenetic allometry, samples should include both juveniles and adults.

## 5 Conclusions

For the analysis of body condition in a single dataset, we expect the results from applying the SMI or other approaches, such as OLS residuals or the Fulton index, to be largely equivalent [46]. For comparisons of results among populations and seasons in the same population, using the SMI with a standard coefficient appears to be the best approach. In studies monitoring green toads, which are of great concern for conservationists in many European countries [47], we propose to calculate the SMI with our ‘green toad coefficient’ (with the formula: mi = m × (60/L)^3^, where mi is the (scaled) mass index (SMI), m is the individual mass, and L is the SVL in mm). Of course, researchers may have good reasons to adopt a different exponent if the data indicate another scaling relationship intheir study populations. In any case, we urge all authors applying the SMI to report the scaling exponent and reference length they used.

## Supplementary Material

**Supplementary Materials:** The following supporting information can be downloaded at: https://www.mdpi.com/article/10.3390/d15010043/s1, Figure S1: Mass versus lengths of toads across all sites with sex specific data, suppl.RCode: RCode_example.zip.

Supplementary Material

## Figures and Tables

**Table 1:** Summary table of the results for each population. Shown are the location-specific parameters and population-specific coefficient, as well as the means and ranges toad measurements. The meanand standard deviation (SD) of the scaled mass index (SMI) calculated using the population-specific coefficient (SMI pop.specific),as well as the SMI using the overall coefficient (SMI.gt), are also shown.

Population	Latitude	Longitude	Elevation	Sample Size (n)	Population-Specific Coefficient	Length in mm (Mean)	Length in mm (Range)	Mass in g (Mean)	Mass in g (Range)	SMI Pop. Specific (Mean)	SMI Pop. Specific (SD)	SMI.gt (Mean)	SMI.gt (SD)
FehmarnIsland (DE)	54.4518	11.0426	1	19	2.349	73.8	62–95	37.5	27.2–70.6	22.8	1.9	20.0	2.3
Warsaw (PL)	52.2676	21.0008	92	78	3.128	65.7	51–81	29.0	13.7–61.4	21.4	2.8	21.6	2.8
EsserKieswerke (DE)	51.0113	6.8690	39	12	3.233	62.0	54.8–66.2	21.8	15.7–27.1	19.5	1.5	19.6	1.4
Ginsterpfad (DE)	50.9858	6.9315	42	20	2.763	65.3	58.3–73.6	25.3	19.6–34.2	19.8	2.0	19.5	2.0
Westhoven (DE)	50.9030	7.0106	45	24	2.747	66.2	44.4–82.7	28.9	11.93–66.4	21.0	3.5	20.4	4.1
Porz-Wahn (DE)	50.8631	7.0896	54	23	4.198	65.6	56.1–76.6	25.4	15.9–38.63	17.3	3.2	19.2	3.3
Basell (DE)	50.8591	6.9485	46	33	3.559	63.2	34.6–72.2	21.9	3.95–31.9	17.7	2.6	18.2	2.0
Urmitz (DE)	50.4081	7.5251	66	209	2.973	58.5	41–76	20.2	6.6–64.2	20.7	3.1	20.7	3.1
Donaufeld (AT)	48.2499	16.4196	161	68	2.689	69.8	40–86	41.2	8.5–73.2	27.1	3.7	25.9	3.7
BednarPark (AT)	48.2258	16.3972	161	178	2.853	58.0	23–78	23.6	1.4–63.4	24.4	3.9	24.4	4.0
Simmering (AT)	48.1690	16.4454	156	840	2.816	61.1	30–84	22.4	3.5–54.56	20.8	3.5	20.7	3.7
Jesenwang (DE)	48.1680	11.1551	556	131	3.522	61.8	48–83	27.5	14–69	24.1	3.2	24.4	3.1
Riem (DE)	48.1336	11.7000	527	110	3.169	27.4	11.25–81	6.8	0.11–65.4	26.8	3.0	22.8	3.4
Seewinkel (AT)	47.7680	16.7866	116	100	2.956	65.6	55–81	30.3	17.5–49.5	22.8	3.2	22.7	3.3
Hochriesgebiet (DE)	47.7465	12.2608	1186	21	2.852	76.6	67–91	47.6	30.5–87.2	23.3	3.3	22.5	3.2
Plovdiv (BG)	42.1573	24.7433	164	61	3.230	67.3	49–79.15	29.6	9–53	21.0	5.1	21.6	5.1
Galabovo (BG)	42.1378	25.8670	96	269	2.042	71.6	61.33–81.12	41.1	30.58–53.22	28.6	2.4	24.3	3.0

## Data Availability

Data are available on reasonable request.
