# Supplementary material for: A Unified Approach to Analysis of Body Condition in Green Toads
Source: Diversity (Basel). Author manuscript; Available in PMC 2023 Mar 29. (PMC7614385; doi:10.3390/d15010043)
Supplement: Supplementary Material [file EMS172738-supplement-Supplementary_Material.zip › Supplement/Supplementary_figure.pdf]

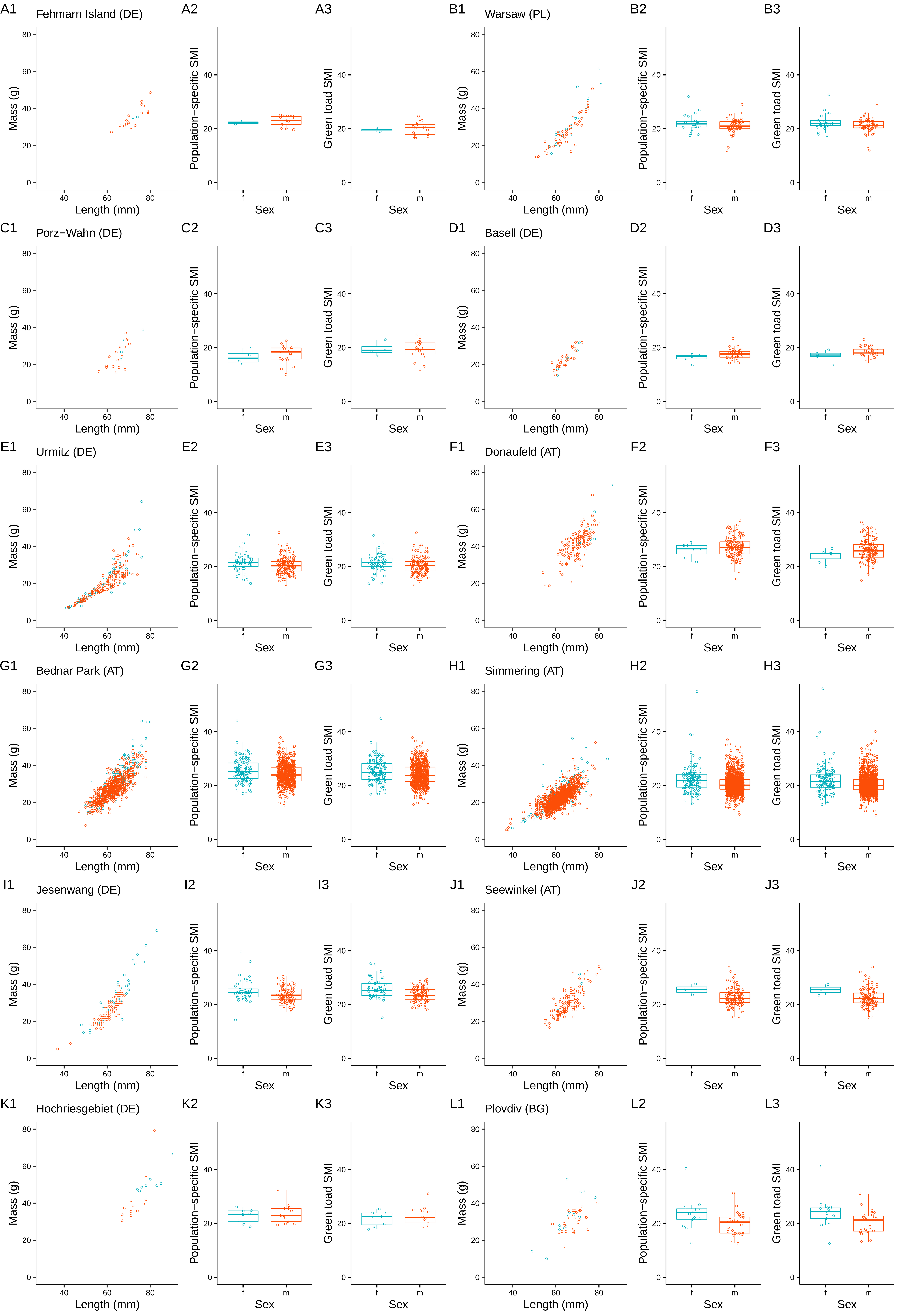

Supplementary Figure: Mass versus lengths of toads (1) as well as the scaled mass index using the population specific coefficient (2) and the green toad coefficient (3) of the populations in Fehmarn Island (A), Warsaw (B) Porz−Wahn (C), Basell (D), Urmitz (E), Donaufeld (F), Bednar Park (G), Simmering (H), Jesenwang (I), Seewinkel (J), Hochriesgebiet (K), Plovdiv (L).
